# Factors influencing health workers’ compliance with the WHO intermittent preventive treatment for malaria in pregnancy recommendations in the Northern Region, Ghana

**DOI:** 10.1186/s12936-022-04286-4

**Published:** 2022-09-24

**Authors:** Abdul Gafaru Mohammed, Dwomoh Duah, Ernest Kenu, Justice Nonvignon, Alex Manu, Harriet Affran Bonful

**Affiliations:** 1 Department of Epidemiology and Disease Control, School of Public Health, University of Ghana, Legon, Ghana; 2 Department of Biostatistics, School of Public Health, University of Ghana, Legon, Ghana; 3 Department of Health Policy, Planning and Management, School of Public Health, University of Ghana, Legon, Ghana

**Keywords:** Compliance, Health workers, IPTp-SP, Ghana, Northern Region, Malaria, Adherence

## Abstract

**Background:**

Although IPTp-SP is a lifesaving World Health Organization (WHO) recommended preventive intervention for pregnant women in malaria-endemic regions, IPTp-SP uptake in the Northern region of Ghana is much lower than the sub-optimal national coverage level. Assessing the extent of health workers’ compliance and its associated factors will generate valuable pointers to be targeted at the program level. The study examined the factors influencing health workers’ compliance with the WHO recommended guidelines for IPTp-SP in the Northern Region.

**Methods:**

A cross-sectional study among 315 health workers in the Northern region was conducted. Semi-structured questionnaires were used to collect data on health workers’ sociodemographic characteristics, facility-based factors and knowledge level. Data were collected on health workers’ compliance with the recommended practices through covert observations using a checklist. Facility observations were carried out using a checklist. Crude and adjusted logistic regression were used to determine predictors of health workers’ compliance, at a 5% significance level adjusting for clustering.

**Results:**

Of the 315 health workers studied, the median age was 29 years (26–34 years). Females constituted (80.5%; 252) of the 313 workers. The majority (47.4%;148) of the 312 health workers were midwives. Overall, 56.2% (CI 51.0 – 62.0) were adequately complying with the recommended guidelines. Lower levels of compliance were recorded in health centres 15.6% (5.0 – 33.0) and CHPS compounds 21.2% (11.0 – 35.0). The factors associated with compliance included health workers’ knowledge (aOR = 7.64, 95% CI 4.21 – 13.87, p < 0.001), job satisfaction (aOR 10.87, 95% CI 7.04 – 16.79, p < 0.001), in-service training (aOR 10.11, 95% CI 4.53 – 22.56, p < 0.001), supervision (aOR 4.01, 95% CI 2.09 – 7.68, p < 0.001), availability of job aids (aOR 3.61, 95% CI 2.44 – 5.35, p < 0.001), health workers experience (aOR = 10.64, 95% CI 5.99 – 18.91, p < 0.001) and facility type (aOR 0.03, 95% CI 0.01–0.07, p < 0.001).

**Conclusion:**

Compliance with the recommended IPTp-SP guidelines is suboptimal in the region, with lower-level health facilities recording the least compliance levels. Health centres and CHPS facilities should be prioritized in distributing limited resources to improve health worker quality of care for antenatal care clients.

**Supplementary Information:**

The online version contains supplementary material available at 10.1186/s12936-022-04286-4.

## Background

Malaria in pregnancy is a significant public health problem. In 2018, an estimated 11 million pregnant women living in 38 countries with moderate-to-high malaria transmission in Sub-Saharan Africa were diagnosed with malaria [1]. Malaria in pregnancy accounts for 16.8% of all hospital admissions and 3.4% of deaths, making it the singular highest contributor to all OPD admissions among pregnant women in Ghana [2, 3].

To reduce the morbidity and mortality associated with malaria, the World Health Organization (WHO) recommended the use of the Intermittent Preventive Treatment for malaria in pregnancy using sulfadoxine-pyrimethamine (IPTp-SP) in 2000, alongside other preventive and curative interventions. The IPTp-SP protects between 65 and 85% of pregnant women from placental malaria infection [4]. The WHO updated the recommendations for IPTp-SP in 2012. The updated three or more doses of IPTp-SP has been demonstrated to be associated with an enhanced reduction in maternal parasitaemia, malaria-related maternal anaemia and poor birth outcomes [5–10]. According to the updated guideline, IPTp-SP should be commenced early in the second trimester, with doses given at least one month apart under direct observation until delivery [6]. Subsequently, the Ghana National Malaria Control Programme revised the national IPTp-SP policy from up to three doses of IPTp-SP to three or more doses until delivery in 2014 [11]. The three or more IPTp-SP doses are linked to a greater mean birth weight and fewer low birth weight (LBW) newborns than the two doses [12].

However, two-thirds of pregnant women do not receive the recommended IPTp-SP doses during their visits to ANC units in Africa [13]. Data from cross-sectional surveys demonstrate that despite the availability of SP, eligible women accessing ANC services in the Volta and Northern regions of Ghana were not offered SP dose [14, 15]. Additionally, although the national target for IPTp-SP3 uptake is 80.0%, data from the District Health Information Management System II (DHIMS II) at the national level indicates that IPTp-SP3 uptake has been below 49% since 2017 [16, 17]. In fact, the Northern region of Ghana recorded a more significant decline in uptake from 36.4% in 2019 to 27.2% in 2020 [16].

Health workers’ inappropriate delivery of IPTp-SP services is a threat to reducing maternal malaria, maternal deaths, and poor birth outcomes. According to the WHO, an essential factor for the low uptake of IPTp-SP3 among pregnant women is confusion among health workers about the IPTp-SP recommendations [6]. In a national survey conducted in 2019, the commonly cited reasons for which 39.0% of pregnant did not receive the required dose during their most recent pregnancy were that they were not aware they had to take more (42%), and health workers did not give it to them (35%) [18].

A combination of interventions is required to improve health workers’ compliance with the WHO IPTp-SP recommendation. These include; regular in-service training, monitoring, supportive supervision, career development programs, providing logistics (portable water, cups for Direct Observation Therapy (DOT practice, SP drug, and IPT manuals or leaflets). Reducing staff workload and motivating staff are equally important conditions worth addressing [15, 19, 20].

Measuring health workers’ compliance with the revised IPTp-SP guidelines and the associated factors will enhance the early identification of areas that need the attention of policymakers and implementors. Researchers can create a composite compliance indicator using 7 binary questions-items [19]. In an attempt to examine the problem of poor compliance to the recommended IPTp-SP guidelines in Ghana, most of the studies considered less than the ideal number of question-items of the IPTp-SP guideline in assessing health workers compliance, potentially leading to an underestimation of compliance level and thus masking the actual burden [15, 20, 23]. Therefore, this study sought to generate data on health workers compliance considering all components of the IPTp-SP guideline and the factors associated in the Northern region.

## Methods

### Study design and setting

A cross-sectional study was conducted among antenatal health workers in the Northern Region of Ghana from April to July 2021. The Northern Region is one of the sixteen regions of Ghana. The annual temperature range is between 25 and 30 °C, which is favourable for *Anopheles* larval development. The region has 308 registered health facilities offering antenatal care services. The region has an estimated 3346 registered community health nurses, enrolled nurses, professional nurses and midwives. Each month, hospitals and polyclinics obtain their supplies of sulfadoxine-pyrimethamine for IPTp administration from the Northern Regional medical stores at no cost. Health centres and Community-based Health Planning and Services (CHPS) compounds at the district level depend on the district medical stores for SP supplies. According to the 2019 Malaria Indicator Survey, 64.5% of pregnant women in the region received three or more doses of IPTp-SP between 2017 and 2019, although 91.8% ANC attendance was recorded [18].

### Study eligibility and sampling process

ANC staff who had worked for at least one year at the selected health facilities and were present at the study time were eligible. Those who had worked for at least one year but were not directly involved in IPTp-SP administration were excluded.

A multistage sampling approach was used in the sampling of 286 health workers. The first stage involved a simple random sampling of 8 districts from the 16 districts in the region. The next stage involved using a restricted stratified sampling approach to select 16 health facilities from the sampled eight districts. In each district, the health facilities were divided into stratum A (district hospitals) and stratum B (Community-based Health Planning and Services (CHPS) compounds, health centres, polyclinics). One facility was selected from each stratum, making up the two health facilities in the district. The last stage involved sampling of 286 health workers from the 16 health facilities sampled. A proportionate to size sampling approach was used to determine the number of health workers to sample from each health facility. Simple random sampling was then used to sample health workers at the various facilities on survey days.

### Data collection and study variables

Data was consecutively collected in two phases. During the first phase, semi-structured questionnaires were administered to the sampled health workers. The questionnaire collected information on health workers’ sociodemographic and individual-based factors. Knowledge was measured using an 11 question item tool for assessing health workers knowledge by Oluwasomidoyin, Bello, & Oni, [29] (Additional file 1). The second phase involved the collection of data on the primary outcome; health workers compliance with the revised IPTp-SP guidelines. This data was collected by covertly observing how health workers performed 7 activities prescribed in the revised guidelines (Additional file 2). Each research assistant was responsible for observing the IPTp-SP practices of their respective respondents. The research assistants observed each of the respondents as they administered the services to their clients. Appropriate safeguards were kept in place allowing research assistants to intervene when necessary to ensure no harm befell the clients in the process of SP administration by the health worker. For example, the research assistants were trained on how to intervene such that the client will not notice the error and lose confidence in the health worker’s abilities, when they observed that a drug other than SP was being administered to a client as SP. No such intervention was necessitated throughout the data collection period though. Research assistants were also trained to replace health workers who noticed they were being observed, however, no replacement was made throughout the data collection period.

Upon completion of observations in each facility, which lasted between 2 and 5 days, research assistants provided feedback to ANC unit in-charges on observed SP administration practices. To safeguard the health workers, feedback given to the ANC unit in charges were generalized without referring to specific health workers. Also, the research team made an agreement with the unit in charges not to punish any of the staff due to the feedback but to put in other measures to improve staff performance. Finally, using a checklist, each health facility was assessed for the availability of SP, portable water and IPTp-SP posters (Additional file 3).

### Data management and statistical analysis

The returned questionnaires were cleaned and edited to ensure accuracy and completeness before coding in Microsoft Excel 2017 and analyzing using STATA version 15.0 (StataCorp LLC). Fifteen forms with missing data on the primary outcome were excluded from the analyses. Regarding the dependent variable, a correct practice was scored 1, and a wrong practice scored 0. The total scores obtained by each health worker were categorized into inadequate compliance (0–3) and adequate compliance (4–7) [19]. Each correct response to the knowledge question items was scored 1, and an incorrect response scored 0. The scores were categorized into low knowledge (0–4), moderate knowledge (5–8) and high knowledge (9–11) [29]. All categorical variables comprising age, sex, cadre, level of education, length of practice, awareness and level of knowledge, type of facility, monitoring and supervision, staff workload, routine training, IPT job aids and manuals, and staff motivation were analysed into frequencies and proportions at 95% CI. Skewed continuous variable, for example, age was presented using a median and interquartile range.

Associations between the independent variables and compliance were tested using crude logistic regression analyses.Variables with statistically significant associations with the health workers’ compliance at a significance level of 0.1 were further selected for adjusted logistic regression analyses. Robust standard errors were used to adjust for clustering in both the crude and adjusted analyses, with the type of facility as the main clustering variable. A significance level of 5% was set for the adjusted logistic analysis. Findings from the health facility assessments were analysed into frequencies and proportions.

## Results

### Health workers background characteristics

Of the 315 health care workers studied, more than two thirds (80.5% ; 252) were females. The median age of the health workers was 29 years (26 – 34 years). On education, 149 (48.7%) of the 306 health workers studied were diploma holders, whiles 53 (17.3%) were degree holders. The majority (59.2%; 177) of the 299 health workers studied had been providing obstetric care for less than five years (Table 1).

**Table 1:** Background Characteristics of Health Workers studied, Northern Region, 2021

Characteristics	Frequency (N = 315)	Percentage (%)
Sex		
Male	61	19.5
Female	252	80.5
Subtotal	313	100.0
Experience(years)		
<5	177	59.2
5 – 10	72	24.1
10 +	50	16.7
Subtotal	299	100.0
Cadre		
General nurse	124	39.7
Midwife	148	47.5
Others	40	12.8
Subtotal	312	100.0
Education		
Certificate	104	34.0
Diploma	149	48.7
Degree	53	17.3
Subtotal	306	100.0
	Median (years)	IQR (years)
Age of HWs	29.0	26.0–34.0

### Health workers’ compliance with the IPTp-SP recommended treatment guidelines

The majority (231; 73.3%) of the 315 health workers properly documented the IPTp-SP services rendered in patients’ ANC cards. Also, most (228; 72.4%) of the 315 health workers studied did not ask if pregnant women were on other medications for example co-trimoxazole. Majority (179; 56.8%) of the 315 health workers failed to inform the pregnant women about the next dose’s visit. (Table 2).

**Table 2:** Observation of Antenatal Health Workers IPTp-SP practices, Northern Region, 2021 (N = 315)

Compliance to IPTp treatment guidelines	Frequency (N)	Percentage (%)
Confirmed gestational period		
Yes	210	66.7
No	105	33.3
Report adverse reactions		
Yes	110	34.9
No	205	65.1
Proper documentation		
Yes	231	73.3
No	84	26.7
DOT practice		
Yes	277	87.9
No	38	12.1
Other medications (co-trimoxazole)		
Yes	87	27.6
No	228	72.4
Required dosage		
Yes	306	97.1
No	9	2.9
Next dose		
Yes	136	43.2
No	179	56.8

### Stratification of health workers’ compliance level by health facility type

Overall, the majority 56.2% (CI 51.0 – 62.0) of the health workers complied with the recommended IPTp-SP practices adequately. However, lower levels of compliance were recorded in the health centres and CHPS compounds (Table 3).

**Table 3:** Stratification of Health Workers’ Compliance level by Health Facility type, Northern Region, 2021

Facility type	Compliance level (%)	95% CI
CHPS	21.2	11.0–35.0
Health Centre	15.6	5.0–33.0
Hospital	69.7	63.0–76.0

### Association between health worker-related factors and their compliance with IPTp-SP guidelines

From the multivariable regression analysis, health workers’ experience, knowledge and use of manuals from personal sources were significantly associated with their compliance with the WHO IPTp-SP guidelines. Health workers who personally sourced and read IPTp-SP manuals had 1.9 times odds of compliance with the recommended guidelines compared to their counterparts (aOR = 1.89, 95% CI 1.04 – 3.43, p < 0.036). Similarly, the odds of complying with the recommended guidelines was 2.8 times among health workers with 5 –10 years working experience (aOR = 2.78, 95% CI 1.06 – 7.24, p < 0.037) and 10.6 times among those with more than ten years of working experience (aOR = 10.64, 95% CI 5.99 – 18.91, p < 0.001) compared with health workers with less than 5 years working experience.

Regarding the knowledge level of the health workers, the odds of complying with the recommended guidelines was 3.2 times among health workers with moderate knowledge compared to those with low knowledge (aOR = 3.20, 95% CI 2.28 – 4.49, p < 0.001). Also, the odds of complying with the recommended guidelines was 7.6 times among health workers with high knowledge compared to those with low knowledge of the guidelines (aOR = 7.64, 95% CI 4.21– 13.87, p < 0.001) (Table 4).

**Table 4:** Association between Health worker-related factors and their Compliance with IPTp-SP guidelines in the Northern Region

Variable	cOR (95% C.I)	P-value	aOR (95% C.I)	P-value
Age of HWs	1.11 (1.1 1.16)	0.001	0.99 (0.96 1.01)	0.325
Sex				
Male	1.00			
Female	0.85 (0.48 1.51)	0.586	0.52 (0.15 1.79)	0.302
Experience				
< 5	1.00			
5–10	3.23 (1.78 5.85)	0.001	2.78 (1.06 7.24)	0.037^++^
> 10	5.65 (2.59 12.33)	0.001	10.64 (5.99 18.91)	0.001^++^
Cadre				
General Nurse	1.00			
Midwife	1.66 (1.02 2.68)	0.041	0.15 (0.06 0.37)	0.001
Others	1.98 (0.95 4.15)	0.070	1.34 (0.65 2.78)	0.429
Education				
Certificate	1.00			
Diploma	1.36 (0.82 2.24)	0.232	2.37 (0.99 5.68)	0.052
Degree	3.69 (1.74 7.81)	0.001	2.27 (0.71 7.26)	0.165
Awareness				
Not Aware	1.00			
Aware	1.51 (0.91 2.48)	0.108	2.56 (0.86 7.78)	0.092
Knowledge level				
Low	1.00			
Moderate	2.90 (1.17 7.17)	0.021	3.20 (2.28 4.49)	0.001^++^
High	9.21 (3.54 23.96)	0.001	7.64 (4.21 13.87)	0.001^++^
Access to IPTp materials				
Not Accessed	1.00			
Accessed	2.21 (1.39 3.51)	0.001	1.89 (1.04 3.43)	0.036^++^

+  + statistically significant

### Association between facility-based factors and health workers’ compliance level

Facility type, job training, staff motivation, supervision and availability of IPTp-SP job aids and manuals were significantly associated with health workers compliance. Health workers who received in-service training on the revised IPTp-SP guidelines had 10.0 times odds of complying with the guidelines compared with those who had not received any training on IPTp-SP administration (aOR 10.11, 95%CI 4.53 – 22.56, p < 0.001).

The odds of compliance among health workers satisfied with the ANC unit's conditions were 10.9 times compared with those not satisfied with the conditions of their working environment (aOR 10.87, 95% CI 7.04 –16.79 p < 0.001).

Similarly, health workers who had been supervised had 4.0 times odds of compliance with the recommended practices than those who had not received any supervisory visits (aOR 4.01, 95% CI 2.09 – 7.68, p < 0.001). Health workers who received IPTp-SP training manuals from their facilities had 3.6 times odds of compliance compared with their counterparts who did not receive these materials from their facilities (aOR 3.61, 95% CI 2.44 – 5.35, p < 0.001) (Table 5).

**Table 5:** Association between facility-based factors and health workers’ Compliance with IPTp-SP guidelines, Northern Region

Variable	cOR (95% CI)	P-value	aOR (95% CI)	P-value
Facility type				
Hospital	1.00			
Health Centre	0.08 (0.03 0.22)	0.001	0.03 (0.01 0.07)	0.001^++^
CHPS compound	0.11 (0.06 0.24)	0.001	0.03 (0.02 0.05)	0.001^++^
Staff workload				
0–30	1.00			
> 30	0.95 (0.60 1.52)	0.829	–	–
Training				
No	1.00			
Yes	13.11 (7.60 22.62)	0.001	10.11 (4.53 22.56)	0.001^++^
Monitoring				
No	1.00			
Yes	16.63 (9.21 30.02)	0.001	4.01 (2.09 7.68)	0.001^++^
Job satisfaction				
Not satisfied	1.00			
Satisfied	12.24 (7.04 21.26)	0.001	10.87 (7.04 16.79)	0.001^++^
IPT training manual				
Not provided	1.00			
Provided	4.50 (2.55 7.95)	0.001	3.61 (2.44 5.35)	0.001^++^
Shortage of SP				
No	1.00			
Yes	0.42 (0.25 0.71)	0.001	1.08 (0.71 1.64)	0.730

+  + statistically significant

### Health facility IPTp-SP implementation assessment

Health education programs on malaria in pregnancy were available in 75.0% (12/16) of the facilities visited. The majority 62.5% (10/16) of health facilities visited had their quarterly education programs, including IPTp-SP. Health talks were delivered in 8 of the 16 health facilities visited. Of these eight facilities, five included malaria in pregnancy in their presentation, whereas only three mentioned IPTp-SP in their presentation. SP was available in all the facilities visited, with eight facilities having posters of IPTp-SP pasted on the walls of the ANC unit. DOT  was practiced in all the facilities visited; however, only two of these health facilities had potable water available for pregnant women. Pregnant women bought water from a seller in the ANC unit or a nearby provision store in the remaining 14 health facilities.

## Discussion

The study indicates that health workers’ experience, knowledge level, training on IPTp-SP, job satisfaction, monitoring, type of facility they work in, and availability of IPTp-SP Job aids were significantly associated with health workers’ appropriate delivery of IPTp-SP. This study used covert observation design similar to that used in other similar studies [26, 27]. The covert participant observation design can provide access to behaviors or practices, or parts of organizations, that would otherwise remain inaccessible to overt research [28]. Covert observation allows the identification of health workers actual compliance with the IPTp-SP recommended guidelines and also minimizes the possibility of Hawthorne’s effect and other biases which are present in overt observations [27, 29]. Covert observation studies have proven beneficial in various health workers compliance studies including compliance to handwashing techniques and other recommended guidelines [28].

Overall, low compliance by health workers recorded at all health system levels was more pronounced in the health centres and CHPS compounds. These CHPS compounds and health centres serve as the first point of health care for most pregnant women in the villages. If not addressed, the high inappropriate delivery of IPTp-SP services will curtail the impact of all demand-side related measures taken to address the low uptake of IPTp-SP in the region. More cost-effective measures adopted to address low compliance in the region should be targeted at the health centres and CHPS compounds. The observed high inadequate compliance among health workers from the CHPS compounds and health centres corroborates the results of a study in the Volta region of Ghana, where inappropriate delivery of IPTp-SP and ITN services were recorded in all facilities studied and more pronounced in the lower level of health care [14]. The consistency in findings could be attributed to the use of observations to assess IPTp-SP delivery in both studies though, De-gaulle & Kamgno used a three-indicator algorithm to assess health workers’ compliance compared to the seven-indicator algorithm employed in the present study.

In-service training of antenatal health care workers keeps them informed and technically equipped to deliver IPTp-SP services to pregnant women. Despite its relevance, training is sometimes overlooked, affecting health workers’ perception of IPTp-SP and its administration. The increased odds of compliance among trained health workers in this study further substantiate the relevance of training to frontline health workers. The significance of training on health workers’ compliance with appropriate practices has also been revealed in other studies among African countries [30, 31]. To ensure adequate training of health workers, the NMCP needs to move from the cascade form of training and adopt a more peripheral system of training where health workers at the health centres and CHPS compounds benefit from the training. Also, the NMCP should adopt adult learning approaches such as table top discussions or functional simulation exercises and feedback delivery to improve health workers adherence to the recommended guidelines [32, 33].

Supervision and monitoring are necessary to keep frontline health workers on their toes and to make them comply with the recommended guidelines. Health workers who had been supervised had an increased odds of compliance with the recommended guidelines. The finding is consistent with a study conducted in Uganda where health workers’ uncertainty of SP and the new IPTp-SP guidelines was associated with a lack of supervision [34]. This implies that an increase in supervision might  improve  the appropriate delivery of IPTp-SP services in the region. The Onsite Training and Supportive Supervision (OTSS) adopted by the National Malaria Control Programme and IMPACT MALARIA for other malaria control areas such as case management and laboratory diagnosis could be extended to the delivery of IPTp-SP services.

Nevertheless, in a study conducted by Maheu-Giroux & Castro [31], health care staff supervised during the six months prior to the study were less likely to deliver IPTp during consultations than those not supervised. The study, unlike this one adopted a secondary data analysis design, which could have accounted for the inconsistency in findings. Health workers access to IPTp training manuals and job aids keep them informed on relevant changes made to existing guidelines. Heath workers with access to job aids and training materials had increased odds of complying with the recommended guidelines. This is similar to the findings of a study conducted among five African countries where IPTp guidelines at health facilities was a significant determinant of IPTp-SP appropriate delivery [34].

Aside supervision another way of ensuring a reduction in deviation of health workers from laid down guidelines is through the empowerment of pregnant women with information to enable them to make demands and or negotiate for the right product or service or procedure to be used [34, 35]. Management of ANC units should promote education of pregnant women about their rights to advocate and negotiate for the right product or service when they perceive health workers could be going wrong.

Health workers are supposed to be motivated to perform IPTp-SP services to the best of their abilities. However, motivating health workers has not been given the needed attention it deserves as a factor that can influence all forms of health service deliveries. In a study conducted in Tanzania, about 80.0% of health workers expressed dissatisfaction as a constrain to their performance [35]. Health workers who were satisfied with the ANC unit’s conditions had increased odds of compliance. Incentivizing best performing health workers at the ANC units of health facilities will motivate health workers to deliver IPTp-SP services.

A few limitations were however identified. The study used covert observation to determine whether health workers were complying with all indicators of the recommended guidelines. One indicator, the proper documentation of services rendered could not be reported based on the observation in a few cases. Research assistants engaged ANC clients to confirm proper IPTp-SP services in their ANC booklets to curtail this limitation. Before the actual data collection, the research assistants received thorough training on data collection tools and extensive pre-test activities to ensure that they were able to gather the data accurately by observing and listening intently to the delivery of care by ANC health workers. Another study limitation was the relatively wide confidence intervals at the health centre level analysis, which could be attributed to the few study participants recruited from health centres using the sampling approach proportionate to size. Caution must be applied in the interpretation thereof about compliance at the health facility level.

## Conclusion

Compliance with the recommended IPTp-SP guidelines is suboptimal in the region, with lower health facilities recording the least compliance levels. Health centres and CHPS facilities should be prioritized in the distribution of limited resources to improve health worker quality of care for antenatal care clients.

## Supplementary Information

**Additional file 1.** Questionnaire on factors influencing health workers compliance with the WHO IPTp-SP recommendation, Northern region, Ghana.

**Additional file 2.** Checklist for health worker IPTp-SP compliance with WHO IPTp-SP treatment guidelines.

**Additional file 3.** Facility assessment.

## Data Availability

The datasets used and/or analyzed during the current study are available from the corresponding author on reasonable request.

## Abbreviations

ANC: Antenatal Care
CHPS: Community based Health Planning and Services
DHIMS: District Health Information Management System
DOT: Direct Observation Therapy
GFELTP: Ghana Field Epidemiology and Laboratory Training Programme
IPTp-SP: Intermittent Preventive Treatment for Malaria in Pregnancy using Sulfadoxine Pyrimethamine
NMCP: National Malaria Control Programme
OPD: Outpatient Department
OTSS: Onsite Training and Supportive Supervision
WHO: World Health Organization

## References

[1] WHO. The “World malaria report 2019” at a glance. Geneva, World Health Organization, 2019. https://www.who.int/news-room/feature-stories/detail/world-malaria-report-2019. Accessed 17 Oct 2020.

[2] NMCP. MEDBOX. 2014 Annual Report: National Malaria Control Programme. 2014. https://www.medbox.org/document/2014-annual-report-national-malaria-control-programme#GO. Accessed 13 Oct 2020.

[3] Owusu-Boateng I, Anto F (2017). Intermittent preventive treatment of malaria in pregnancy: a cross-sectional survey to assess uptake of the new sulfadoxine-pyrimethamine five dose policy in Ghana. Malar J.

[4] Garner P, Gülmezoglu AM (2006). Drugs for preventing malaria in pregnant women. Cochrane Database Syst Rev.

[5] Amoakoh-Coleman M, Arhinful DK, Klipstein-Grobusch K, Ansah EK, Koram KA (2020). Coverage of intermittent preventive treatment of malaria in pregnancy (IPTp) influences delivery outcomes among women with obstetric referrals at the district level in Ghana. Malar J.

[6] WHO Global Malaria Programme. New WHO recommendations for IPTp-SP. Geneva, World Health Organization, 2013. http://whqlibdoc.who.int/publications/2010/9789241599412_eng.pdf. Accessed 17 Oct 2020.

[7] Orobaton N, Austin AM, Abegunde D, Ibrahim M, Mohammed Z, Abdul-Azeez J (2016). Scaling-up the use of sulfadoxine-pyrimethamine for the preventive treatment of malaria in pregnancy: results and lessons on scalability, costs and programme impact from three local government areas in Sokoto State. Nigeria Malar J.

[8] Dapaa S. Uptake of intermittent preventive treatment for malaria and birth outcomes in selected health facilities in the Brong Ahafo Region of Ghana. MPhil Thesis, University of Ghana, 2017;1–77.

[9] Gutman J, Mwandama D, Wiegand RE, Ali D, Mathanga DP, Skarbinski J (2013). Effectiveness of intermittent preventive treatment with sulfadoxine-pyrimethamine during pregnancy on maternal and birth outcomes in Machinga District, Malawi. J Infect Dis.

[10] Martin MK, Venantius KB, Patricia N, Bernard K, Keith B, Allen K (2020). Correlates of uptake of optimal doses of sulfadoxine-pyrimethamine for prevention of malaria during pregnancy in East-Central Uganda. Malar J.

[11] MoH. Management of cases in Ghana. J Ghana Sci*. *2014;11(5):201–42,

[12] WHO Global Malaria Programme. New WHO recommendations for IPTp-SP. 2013. http://whqlibdoc.who.int/publications/2010/9789241599412_eng.pdf. Accessed 23 Oct 2020.

[13] Azizi SC (2020). Uptake of intermittent preventive treatment for malaria during pregnancy with Sulphadoxine-Pyrimethamine in Malawi after adoption of updated World Health Organization policy: an analysis of demographic and health survey 2015–2016. BMC Public Health.

[14] De-Gaulle VF, Magnussen P, Kamgno J, Mbacham W, Orish VN, Tagbor H (2021). Assessing health system factors affecting access and delivery of IPTp-SP and ITN to pregnant women attending ANC clinics in Ghana. BMC Health Serv Res.

[15] Stephen AAI, Wurapa F, Afari EA, Sackey SO, Malm KL, Nyarko KM (2016). Factors influencing utilization of intermittent preventive treatment for pregnancy in the Gushegu district, Ghana, 2013. Pan Afr Med J.

[16] NMCP. 2020 Annual malaria report for Ghana. Accra, 2020.

[17] NMCP. 2017 Annual Report National Malaria Control Programme. Accra, 2017.

[18] MIS. The DHS Program - Ghana: Malaria Indicator Survey (MIS), 2019. https://www.dhsprogram.com/what-we-do/survey/survey-display-557.cfm. Accessed 16 Sep 2020.

[19] Arulogun OS, Okereke CC (2012). Knowledge and practices of intermittent preventive treatment of malaria in pregnancy among health workers in a southwest local government area of Nigeria. J Med Med Sci.

[20] Amankwah S, Anto F (2019). Factors associated with uptake of intermittent preventive treatment of malaria in pregnancy: a cross-sectional study in private health facilities in Tema Metropolis, Ghana. J Trop Med.

[21] Vandy AO, Peprah NY, Jerela JY, Titiati P, Manu A, Akamah J (2019). Factors influencing adherence to the new intermittent preventive treatment of malaria in pregnancy policy in Keta District of the Volta region, Ghana. BMC Pregnancy Childbirth.

[22] Orish VN, Onyeabor OS, Boampong JN, Afoakwah R, Nwaefuna E, Acquah S (2015). Prevalence of intermittent preventive treatment with sulphadoxine-pyrimethamine (IPTp-Sp) use during pregnancy and other associated factors in Sekondi-Takoradi, Ghana. Afr Health Sci.

[23] Ibrahim H, Maya ET, Issah K, Apanga PA, Bachan EG, Noora CL (2017). Factors influencing uptake of intermittent preventive treatment of malaria in pregnancy using sulphadoxine pyrimethamine in sunyani municipality, Ghana. Pan Afr Med J.

[24] Wu KS, Chen YS, Lin HS, Hsieh EL, Chen JK, Tsai HC (2017). A nationwide covert observation study using a novel method for hand hygiene compliance in health care. Am J Infect Control.

[25] Ameme DK, Odikro MA, Baidoo A, Dsane-Aidoo P, Nuvey FS, Jackson DG (2021). Hand hygiene and face mask wearing practices for COVID-19 prevention: a non-intrusive observation of patrons of community convenience shops in Accra, Ghana. Pan Afr Med J.

[26] Petticrew M, Semple S, Hilton S, Creely KS, Eadie D, Ritchie D (2007). Covert observation in practice: lessons from the evaluation of the prohibition of smoking in public places in Scotland. BMC Public Health.

[27] Spicker P (2011). Ethical covert research. Sociology.

[28] Werzen A, Thom KA, Robinson GL, Li S, Rock C, Herwaldt LA (2019). Comparing brief, covert directly-observed hand hygiene compliance monitoring to standard methods: a multicenter cohort study. Am J Infect Control.

[29] Bello OO, Oni O (2020). Health workers' awareness and knowledge of current recommendation of intermittent preventive treatment in pregnancy in south-western Nigeria. Ethiop J Health Sci.

[30] Oyefabi A, Sambo M, Sabitu K (2015). Effect of primary health care workers training on the knowledge and utilization of intermittent preventive therapy for malaria in pregnancy in Zaria, Nigeria. J Med Trop.

[31] Maheu-Giroux M, Castro MC (2014). Factors affecting providers’ delivery of intermittent preventive treatment for malaria in pregnancy: a five-country analysis of national service provision assessment surveys. Malar J.

[32] Aggarwal R, Mytton OT, Derbrew M, Hananel D, Heydenburg M, Issenberg B (2010). Training and simulation for patient safety. Qual Saf Health Care.

[33] Lababidi HMS, Alzoraigi U, Almarshed AA, Alharbi W, Alamar M, Arab AA (2021). Simulation-based training programme and preparedness testing for COVID-19 using system integration methodology. BMJ Simul Technol Enhanc Learn.

[34] Rassi C, Graham K, Mufubenga P, King R, Meier J, Gudoi SS (2016). Assessing supply-side barriers to uptake of intermittent preventive treatment for malaria in pregnancy: a qualitative study and document and record review in two regions of Uganda. Malar J.

[35] Mubyazi GM, Bloch P, Byskov J, Magnussen P, Bygbjerg IC, Hansen KS (2012). Supply-related drivers of staff motivation for providing intermittent preventive treatment of malaria during pregnancy in Tanzania: evidence from two rural districts. Malar J.

