# Supplementary material for: Factors influencing health workers’ compliance with the WHO intermittent preventive treatment for malaria in pregnancy recommendations in the Northern Region, Ghana
Source: Malar J. 2022 Sep 24;21:273. doi: 10.1186/s12936-022-04286-4 (PMC9509592; doi:10.1186/s12936-022-04286-4)
Supplement: Supplementary file 1 — Additional file 1. Questionnaire on factors influencing health workers compliance with the WHO IPTp-SP recommendation, Northern region, Ghana. [file 12936_2022_4286_MOESM1_ESM.docx]

## **QUESTIONNAIRE ON FACTORS INFLUENCING HEALTH WORKERS COMPLIANCE WITH THE WHO IPTp-SP RECOMMENDATION, NORTHERN REGION, GHANA**

**QUESTIONNAIRE NO :**

**INTERVIEWER**

I am a post graduate student from the University of Ghana undertaking a study on health workers Compliance with the WHO IPTp-SP recommendations. I would be glad if you participate in this study by answering a few questions to enable the achievement of the objectives of this study. Your responses would be treated as confidential and used only for the purposes of this research. Your name is not required. Kindly respond as truthfully as possible. You can ask questions if you want.

Thank you.

**Respondent general information**

Name of health facility ………………………………………………

**SECTION 1**

**Health workers demographic information**

| **No** | **Question** | **Response** | **Code** |
| --- | --- | --- | --- |
| Q1 | What is your sex? | Male 1  Female 2 | Q1sex |
| Q2 | What is your age? | ………… | Q2age |
| Q3 | What is your profession? | General Nurse 1  Midwife 2  Others 3  specify…………………… | Q3cadre |
| Q4 | What is your highest educational level? | Certificate 1  Diploma 2  BSC 3  Other, specify………………… | Q4educ |
| Q5 | How many pregnant women do you attend to in a week? | ……………………….. | Q5load |
| Q6 | How long have you worked in the medical field? | ………………………… | Q6experience |

**SECTION 2**

In this section, I would like to ask questions about IPTp and how it is administered to pregnant women

**Awareness & Knowledge of ANC Staffs on Intermittent Preventive Therapy of Malaria Using Sulfadoxine-Pyrimethamine –IPTp-SP**

| **No** | **STAFF KNOWLEDGE ABOUT IPTp-SP** | **Response** | **Code** |
| --- | --- | --- | --- |
| Q7 | What drug is used for malaria prevention during pregnancy? | SP (Sulfadoxine Pyrimethamine) 1  Quinine 2  Other, specify 3 | Q7kw |
| Q8 | What minimum dose of SP is required during the entire pregnancy? | One 1  Two 2  Three 3  More than 3 4  Other, specify…………………… | Q8kw |
| Q9 | How many doses of SP are recommended for a pregnant woman to take during her entire pregnancy? | One 1  Two 2  Three 3  More than 3 4  Other, specify…………………… | Q9kw |
| Q10 | When is the recommended gestation age for the first dose of SP for IPTp? | 12^th^ week. 1  16^th^ week 2  20-24 weeks 3  30-36 weeks 4  Other, specify…………………… | Q10kw |
| Q11 | What is the time interval between one dose of SP and the next dose? | Two-weeks 1  Four-weeks 2  8 or more weeks 3  Don't know 4  Other, specify…………………… | Q11kw |
| Q12 | To your knowledge up to what period can SP be given during pregnancy? | Up to 32 weeks 1  Up to 34 weeks 2  Up to delivery 3  Don't know 4 | Q12kw |
| Q13 | Where is the recommended place for SP to be swallowed? | In front of ANC staff 1  At home 2  On the way home 3  Don't know. 4  Other, specify…………………… | Q13kw |
| Q14 | During which period (GA in weeks) is SP not allowed to be given during pregnancy | <12weeks. 1  16^th^ week 2  20-24 weeks 3  30-36 weeks 4  Other, specify…………………… | Q14kw |
| Q15 | During pregnancy which condition can hinder a pregnant woman from taking SP for IPTp | Diarrhea 1  HIV (co-trimoxazole) 2  Vomiting 3  G6PD 4  Don't know 5  Other, specify…………………… | Q15kw |
| Q16 | If a pregnant woman is diagnosed with malaria during routine ANC attendances, what will you do? | Treat her with SP 1  Withhold SP and allow patient be treated with ALU/Quinine 2  Don't know 3  Other, specify…………………… | Q16kw |
| Q17 | If a pregnant woman who attends ANC is allergic to SP, what will you do for prevention of malaria during pregnancy? | Stop SP completely 1  Give anti allergic drug and give SP 2  Will prescribe another antimalarial drug aside SP 3  Don't know 4  Other, specify…………………… | Q17kw |

**SECTION 3**

**In this section,** I would like to ask questions on your workload, availability of SP stock, available motivating factors, training sessions and supervisory services

|  | **IPTp training manuals and SP availability** | **Response** | **Code** |
| --- | --- | --- | --- |
| Q18 | Does your facility provide you with IPTp training materials | Yes/1  No/0 | Q18sp |
| Q19 | Have you sourced and read any IPTp training material in the last 6 months | Yes/1  No/0 | Q19sp |
| Q20 | Have you experienced a shortage of SP in the last 6 months | Yes/1  No/0 | Q20sp |
|  | **Staff motivation and workload** | **Response** | **Code** |
| Q21 | Averagely how many pregnant women do you attend to in a day | ……………. | |
| Q22 | Are you satisfied with all the working conditions surrounding the ANC unit | Yes/1  No/0 | Q22mt |
|  | **Staff training** | **Response** | **Code** |
| Q23 | Have you had any training in IPTp before | Yes/1  No/0 | Q23tg |
| Q24 | If yes, was it helpful | Yes/1  No/0 | Q24tg |
| Q25 | How many times in the last twelve months did you have training on IPTp? | ………………….. | Q25tg |
|  | **Staff supervision and monitoring** | **Response** | **Code** |
| Q26 | Did you have any supervisory/monitoring visits at your unit last year | Yes/1  No/0 | Q26sm |
| Q27 | If yes, how many times during the last year did you have monitoring/supervisory visits for IPTp? | …………. | Q27sm |
| Q28 | Who did the monitoring/supervision | ………………………. | Q28sm |
