# Supplementary material for: Factors influencing health workers’ compliance with the WHO intermittent preventive treatment for malaria in pregnancy recommendations in the Northern Region, Ghana
Source: Malar J. 2022 Sep 24;21:273. doi: 10.1186/s12936-022-04286-4 (PMC9509592; doi:10.1186/s12936-022-04286-4)
Supplement: Supplementary file 2 — Additional file 2. Checklist for health worker IPTp-SP compliance with WHO IPTp-SP treatment guidelines. [file 12936_2022_4286_MOESM2_ESM.docx]

# CHECKLIST NUMBER

**Checklist for Health Worker IPTp-SP Compliance with WHO IPTp-SP treatment guidelines**

| **No** | **ADHERENCE TO IPTp-SP RECOMMENDATIONS** | **Response** | **Code** |
| --- | --- | --- | --- |
| Q1 | Did HCW confirm the duration of pregnancy | Yes/1  No/0 | Q1ad |
| Q2 | Did she ask if the client is on other any medications such as co-trimoxazole | Yes/1  No/0 | Q2ad |
| Q3 | Did HCW administer the required dosage | Yes/1  No/0 | Q3ad |
| Q4 | Did the client take the drug in front of the health worker | Yes/1  No/0 | Q4ad |
| Q5 | Did HCW record on the ANC booklet and ward register | Yes/1  No/0 | Q5ad |
| Q6 | Did the HCW inform the client on when the next dose is due | Yes/1  No/0 | Q6ad |
| Q7 | Did the HCW inform the client to report adverse reactions | Yes/1  No/0 | Q7ad |
