# Supplementary material for: Factors influencing health workers’ compliance with the WHO intermittent preventive treatment for malaria in pregnancy recommendations in the Northern Region, Ghana
Source: Malar J. 2022 Sep 24;21:273. doi: 10.1186/s12936-022-04286-4 (PMC9509592; doi:10.1186/s12936-022-04286-4)
Supplement: Supplementary file 3 — Additional file 3. Facility assessment. [file 12936_2022_4286_MOESM3_ESM.docx]

## **FACILITY ASSESSMENT**

**Facility general information**

| **No** | **Question** | **Response** | **Code** |
| --- | --- | --- | --- |
| Q1 | Date of Survey |  | Q1date |
| Q2 | code |  | Q2id |
| Q3 | Name of District |  | Q3district |
| Q4 | Name of facility |  | Q4facility |

**Observation of facility practices on IPTp-SP treatment guidelines**

| Q5 | Health education program drawn for the quarter includes MIP | Yes/1  No/0 | Q5ff |
| --- | --- | --- | --- |
| Q6 | Health education program drawn for the quarter includes IPTp | Yes/1  No/0 | Q6ff |
| Q7 | Health talk given at ANC on day of visit | Yes/1  No/0 | Q7ff |
| Q8 | Health talk given that day included malaria in pregnancy | Yes/1  No/0 | Q8ff |
| Q9 | Health talk given that day included IPTp | Yes/1  No/0 | Q9ff |
| Q10 | Presence of posters of IPTp/MIP on the wall | Yes/1  No/0 | Q10ff |
| Q11 | SP available at ANC | Yes/1  No/0 | Q11ff |
| Q12 | Practice of DOT | Yes/1  No/0 | Q12ff |
| Q13 | Presence of free, clean, safe water for DOT | Yes/1  No/0 | Q13ff |
